# Strukturierte Befunderhebung in der Hals-Nasen-Ohren-Heilkunde

**DOI:** 10.1007/s00106-025-01605-4

**Published:** 2025-03-26

**Authors:** Benjamin Philipp Ernst

**Affiliations:** Klinik für Hals-Nasen-Ohrenheilkunde, Universitätsklinikum Frankfurt, Theodor-Stern-Kai 7, 60590 Frankfurt am Main, Deutschland

**Keywords:** Reliabilität und Validität, Algorithmus, Sonographie, Klassifikation, Vertigo, Reliability and validity, Algorithm, Ultrasound, Classification, Vertigo

## Abstract

Die strukturierte Befunderhebung (SR) hat als wertvolle Methode zur Optimierung von Diagnose und Therapie einen zentralen Stellenwert in verschiedenen Fachdisziplinen. Während herkömmliche Freitextbefunde oft uneinheitlich und schwer vergleichbar sind, ermöglicht die strukturierte Dokumentation eine höhere Qualität und Vollständigkeit der Befunde. Die trägt dazu bei, die steigende Komplexität besser zu bewältigen und den therapeutischen Standard zu erhöhen. Studien zeigen, dass SR in verschiedenen Teilgebieten der Hals-Nasen-Ohren-Heilkunde (HNO) zu einer signifikanten Verbesserung der Befundqualität führt. Zudem steigert SR die zeitliche Effizienz sowie die Interrater-Reliabilität und trägt zum Lerneffekt bei. Ferner erhöht SR die Anwender- und Zuweiserzufriedenheit, insbesondere bei interdisziplinärer Anwendung. Zukünftige multizentrische Studien sind notwendig, um weitere Erkenntnisse zur praktischen Anwendung, zur wissenschaftlichen Auswertbarkeit und zur Kombination mit künstlicher Intelligenz liefern.

## Abstract (en)

Structured reporting (SR) is a valuable method for optimizing diagnosis and treatment in various specialist disciplines. While conventional free-text findings are often inconsistent and difficult to compare, structured documentation enables higher quality and completeness of findings. This helps to better manage the increasing complexity and raise therapeutic standards. Studies show that SR leads to a significant improvement in the quality of findings in various areas of otorhinolaryngology. SR also increases time efficiency and inter-rater reliability and contributes to the learning effect. Furthermore, SR increases user and referring physician satisfaction, especially in interdisciplinary application. Future multicenter studies are needed to provide further insights into the practical application and scientific evaluability of SR, also in combination with artificial intelligence.

## Befunderhebung und Dokumentation in der Hals-Nasen-Ohren-Heilkunde

**Abb. 1:**
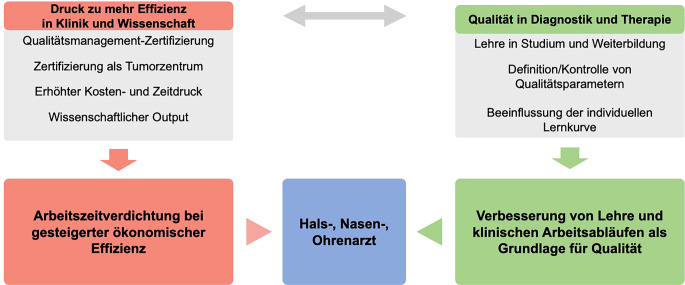
Herausforderungen in der modernen Hals-Nasen-Ohren-Heilkunde

In den vergangenen Jahrzehnten wurden die Ergebnisse von Untersuchungen in der Hals-Nasen-Ohren-Heilkunde (HNO) typischerweise als handschriftliche Freitextbefunde (FTR) festgehalten [30, 101, 102]. Optional werden diese durch Piktogramme bzw. Skizzen ergänzt, in denen Form und Lage etwaiger pathologischer Veränderungen manuell eingezeichnet werden können. Obwohl diese Praxis langfristig in der Routine etabliert ist, ergeben sich hieraus eine Vielzahl von Problemen im klinischen Alltag, da diese Aufzeichnungen als Basis für Kontrolluntersuchungen, Arztbriefe, Abrechnung und die weitere Therapieplanung dienen. Zudem stellt diese klassische Art der Befunddokumentation Mindestanforderungen an die Sorgfalt des befundenden Arztes, sowohl bezüglich seiner Handschrift, seiner Skizzierungen als auch ihrer Vollständigkeit. Insbesondere in der letzten Dekade hat neben einer immensen Arbeitsverdichtung aufgrund des hohen wirtschaftlichen Drucks innerhalb des medizinischen Sektors eine zunehmende Komplexität durch neue, sowohl prognostisch als auch therapeutisch zu berücksichtigende Parameter und Einteilungssysteme Einzug in die HNO gehalten (Abb. 1; [12, 55, 87]). Dies stellt eine große Herausforderung für die studentische und assistenzärztliche Aus- und Weiterbildung dar, da hier oftmals sowohl die zu evaluierenden als auch zu dokumentierenden Strukturen und in der Folge auch die notwendige Terminologie unklar sind [30, 33]. Folge ist eine notwendige und extrem personalintensive Überprüfung der Prozesse durch die verantwortlichen Vorgesetzten und das fortbestehende Risiko, pathologische Veränderungen zu übersehen oder diese unzureichend zu dokumentieren.

Von besonderer Bedeutung sind die beschriebenen Probleme der konventionellen FTR bei der klinischen Verlaufskontrolle verschiedenster pathologischer Veränderungen, beispielsweise von zervikalen Lymphadenopathien, Speicheldrüsenerkrankungen oder Kopf-Hals-Karzinomen, da hier oftmals sowohl die Lokalisation als auch das Ausmaß oder die Form des pathologischen Befundes nur unzureichend beschrieben sein können [1, 6, 56, 97]. Eine gute Lokalisationsdiagnostik stellt jedoch die Grundlage der weiteren – ggf. chirurgischen – Therapieplanung dar und ist damit essenziell wichtig. Gleiches gilt für die Abrechenbarkeit klinischer Leistungen, da nur korrekt dokumentierte klinische Untersuchungen möglichen Überprüfungen der Kostenträger standhalten können [16]. Zusammenfassend eignen sich FTR nur eingeschränkt zur Verwertung für klinische Forschung, da sie teilweise kaum vergleichbar sind und die korrekte Identifikation spezifischer Krankheitsbilder äußerst zeitaufwendig und fehleranfällig ist [81]. Zudem stellen sie keine nutzbare Grundlage für die Etablierung künstlicher Intelligenz (KI) im Rahmen von Large-Language-Modellen (LLM) oder zur Auswertung mittels Big-Data-Analysen dar [52, 81, 113].

## Strukturierte Befunderhebung

### Allgemeine Aspekte

In den vergangenen Jahren ist es zu einem verstärkten Einsatz von diagnostischen und therapeutischen Parametern sowie Staging-Klassifikationen in der HNO gekommen [50, 78, 84, 104]. Dies ermöglicht auf der einen Seite eine deutlich bessere Vergleichbarkeit, hat aber auf der anderen Seite auch zu einer deutlichen Zunahme der Komplexität innerhalb des Fachgebiets geführt. Darüber hinaus ist aufgrund von steigenden Patientenzahlen ohne gleichsinnige Entwicklung der Personalstellenpläne eine vermehrte Arbeitsverdichtung zu verzeichnen. Um dieser steigenden Komplexität gerecht zu werden und um einen höchstmöglichen medizinischen Standard zu gewährleisten, sind möglichst genaue, detaillierte und vergleichbare Befunde von zentraler Bedeutung. Mit dem Ziel, eine qualitativ höherwertige Befundung zu erreichen, plädieren diverse fachspezifische und fachfremde Fachgesellschaften für die Implementierung von digitalen strukturierten Befunderhebungen (SR) [23, 31, 59, 70]. Gemäß den generell akzeptierten Definitionen besteht dabei ein strukturierter Befund u. a. aus standardisierten Überschriften sowie Unterkategorien, die der Spezifizierung von Ergebnissen dienen, und einer standardisierten Terminologie [8, 115]. Weiss und Bolos beschrieben bereits 2009 dabei 3 Level der SR [10, 75]:

1. Strukturiertes Format: Definition von Abschnitten und Unterabschnitten
2. Konsequenter Aufbau des Befundes: Festlegung und Reihenfolge der zu befundenden Strukturen
3. Konsequente Anwendung der vorgesehenen Terminologie (standardisierter sprachlicher Aufbau)

Diese 3 Ebenen der SR nach Weiss und Bolos wurden 2020 von Nobel et al. durch 2 weitere Level ergänzt [75]:

4. Strukturiertes Layout: Darstellung des Befundes in einer strikten, vordefinierten Reihenfolge, um eine Gleichförmigkeit der Befunde zur erreichen bzw. aufrechtzuerhalten
5. Strukturierter Inhalt: festgelegte Art und Weise, in der der medizinische Inhalt des Befundes IT-gestützt angeordnet und im Bericht angezeigt wird

**Abb. 2:**
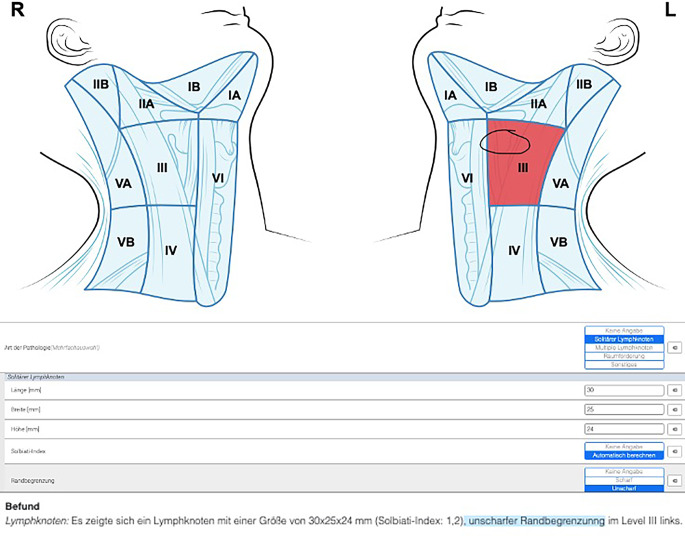
Strukturierte Befunderhebung eines solitären pathologischen Lymphknotens. (Mit freundl. Genehmigung durch Smart Reporting GmbH)

Im Kontext von Big-Data-Analysen würde die Datenspeicherung in strukturierten Speicherelementen gemäß Froelich et al. den abschließenden Schritt der Befundstrukturierung darstellen (Abb. 2; [34]). Dies ermöglicht neben der verbesserten Auswertbarkeit auch die Anwendung von datenschutzrechtlich unkomplizierten lokalen LLM-Lösungen zur Augmentation von klinischen Arbeitsabläufen durch KI [13, 111].

Während konventionelle FTR eine tendenziell niedrige Intra- und Interrater-Reliabilität in Bezug auf Befundqualität, Detailliertheit und Vergleichbarkeit aufweisen, stellen strukturierte Befunde einen vielversprechenden neuen Ansatz in der Befunderstellung dar [30, 76].

So wurde in verschiedenen Studien eine Tendenz zur höherer Befundqualität, Genauigkeit und Detailliertheit im Vergleich zu Freitextbefunden sowie eine deutliche Präferenz der Anwender für SR nachgewiesen [37, 76, 79, 95, 97]. Ferner minimieren strukturierte Befundvorlagen mit standardisierten Kapiteln, Format und Terminologie das Risiko, wichtige Strukturen bei der Untersuchung zu übersehen oder diese adäquat zu beschreiben [51, 79, 84, 110]. Daher erhöht SR insbesondere bei jüngeren Weiterbildungsassistenten die Befundqualität und leistet einen wichtigen Beitrag im Lernprozess [88, 97].

Diese Erkenntnisse könnten in der Konsequenz zu einem besseren Verständnis von pathologischen Veränderungen und deren therapeutischen Implikationen führen [14, 58]. Auf der anderen Seite wird von vielen erfahrenen Diagnostikern häufig kritisiert, dass SR-Vorlagen oftmals zu rigide in ihrer Anwendung und somit möglicherweise unpräzise und zeitaufwendig seien [8, 51]. Folglich eignet sich diese Form der Befunderhebung besonders für Arbeitsabläufe in der HNO mit einem hochgradig standardisierten Arbeitsablauf, wie beispielsweise Ultraschalluntersuchungen der Halsweichteile (Abb. 2), die neurootologische Abklärung von Schwindelbeschwerden oder die Begutachtung von Computertomographien (CT) der Nasennebenhöhlen (NNH). Eine ausführliche Übersicht zur Datenlage zur SR in der HNO sowie zu den entsprechenden Studienendpunkten ist Tab. 1 zu entnehmen.

**Tab. 1:** Vorliegende Evidenz und signifikante Endpunkte für die strukturierte Befunderhebung in der Hals-Nasen-Ohren-Heilkunde

Autor	Titel	Thematik	Signifikante Endpunkte für strukturierte Befunderhebung
Ernst et al. [25]	Structured reporting of head and neck ultrasound examinations	Ultraschall	Befundqualität, Befundvollständigkeit, Benutzerzufriedenheit
Ernst et al. [26]	Impact of structured reporting on developing head and neck ultrasound skills	Ultraschall	Befundqualität, Befundvollständigkeit, Benutzerzufriedenheit, zeitliche Effizienz
Ernst et al. [28]	The use of structured reporting of head and neck ultrasound ensures time-efficiency and report quality during residency	Ultraschall	Befundqualität, Befundvollständigkeit, Benutzerzufriedenheit, zeitliche Effizienz, longitudinaler Lerneffekt
Ernst et al. [29]	Evaluation of optimal education level to implement structured reporting into ultrasound training	Ultraschall	Befundqualität, Befundvollständigkeit, Benutzerzufriedenheit, Zeitpunkt Implementierung
Ernst et al. [27]	The role of structured reporting and structured operation planning in functional endoscopic sinus surgery	Nasennebenhöhlenchirurgie	Vollständigkeit Befund und Operationsplanung, zeitliche Effizienz, Benutzerzufriedenheit
Becker et al. [5]	ENT residents benefit from a structured operation planning approach in the training of functional endoscopic sinus surgery	Nasennebenhöhlenchirurgie	Vollständigkeit der Operationsplanung, zeitliche Effizienz, Benutzerzufriedenheit
Ernst et al. [24]	Structured reporting of head and neck sonography achieves substantial interrater reliability	Ultraschall	Interrater-Reliabilität, Befundvollständigkeit
Lasrich et al. [60]	Erhöhte Befundvollständigkeit und gesteigerte Zuweiserzufriedenheit bei strukturierter neurootologischer Befunderhebung in der interdisziplinären Schwindelabklärung	Neurootologie	Befundvollständigkeit, Benutzerzufriedenheit

#### Anwendungsgebiete

So wurde bereits eine signifikante Überlegenheit von strukturierten Befunden im Rahmen der Videofluoroskopie bei Schluckstörungen, der Magnetresonanztomographie (MRT) der Schulter zur Operationsplanung von Schulterstabilisierungen, der MRT des Beckens zum lokoregionären Staging beim Rektumkarzinom vor Therapie sowie der CT des Thorax bei V. a. Lungenarterienembolie nachgewiesen [37, 76, 95–97]. Neben einer signifikant erhöhten Befundqualität zeigte sich in diesen Studien mittels Fragebögen eine deutliche Präferenz sowohl der Untersucher als auch der Zuweiser bzw. in der Konsequenz der behandelnden Chirurgen. Diese Präferenz für strukturierte Befunde ist insbesondere in der standardisierten SR und Befundstruktur sowie den zugrunde liegenden Leitlinien und klinischen Standards begründet. Darüber hinaus scheint SR Assistenzärzte in der frühen Weiterbildungsphase bei der Erlernung diagnostischer Modalitäten und deren Befundung zu unterstützen [97]. Ferner wird die Lernkurve von unerfahrenen Weiterbildungsassistenten durch Darlegung eines Untersuchungs- bzw. Befundungsalgorithmus und durch Fokussierung auf essenzielle Strukturen innerhalb des Untersuchungsfelds beschleunigt [26, 107, 117]. In der Konsequenz wurde gezeigt, dass mittels SR die Rate an übersehenen pathologischen Veränderungen signifikant reduziert werden und somit die diagnostische Qualität deutlich gesteigert werden kann [51, 63].

Darüber hinaus ergeben sich aus der standardisierten Befundstruktur in der Zukunft Möglichkeiten zur automatisierten Einschätzung des Therapieansprechen oder auch zur Generierung von Daten für wissenschaftliche Analysen [81].

#### Mögliche Problemfelder und Vorteile im klinischen Alltag

Ein kontroverses Thema bei der Erstellung medizinischer Befunde ist, wie bereits erwähnt, ob strukturierte Befunde zu rigide sind und in der Folge die Qualität der Befunde einschränken [8, 51]. Diesen Kritikpunkten kann jedoch durch Zuhilfenahme moderner Softwaresysteme, die mittels intelligenter Entscheidungsbäume mit der Möglichkeit zur Verlinkung ein hohes Maß an linguistischer Qualität garantieren, begegnet werden [75, 94]. Ferner wird durch Verwendung von Freitextfeldern mit der Möglichkeit zur Ergänzung von spezifischen Befundelementen ein Höchstmaß an Flexibilität und Vollständigkeit erreicht [74, 95]. Darüber hinaus ermöglicht eine einmal erstellte Maske zur SR eine nahezu fehlerfreie Befunderstellung in Bezug auf Rechtschreibung und Grammatik. Moderne SR-Softwarelösungen implementieren zudem die Möglichkeit zur Befunderstellung in Fremdsprachen, wodurch auch bei Nichtmuttersprachlern eine maximal hohe Befundqualität erreicht werden kann [103]. Das Problem von Befunden, die durch nichtmuttersprachliches ärztliches Personal erstellt werden, wird durch die zunehmende Bedeutung der Telemedizin unterstrichen [65]. Insbesondere die teleradiologische Befundung ist für ländliche Gebiete mit Fachkräftemangel eine Notwendigkeit geworden, etwa am Beispiel der teleneuroradiologischen Betreuung dezentraler Stroke Units. Dieses Problem kann durch Breitbandverbindungen gelöst werden. Sie ermöglichen die Übertragung großer Datenmengen, die in anderen Regionen, ob im Inland oder im Ausland, beurteilt werden können. Im Fall von telemedizinischen Befundungen im Ausland kann die damit einhergehende Sprachbarriere eine essenzielle Hürde bei der Erstellung qualitativ hochwertiger Befundungen darstellen. Diese sind wiederum mit einer erhöhten Rate an Nachfragen assoziiert, was zur Arbeitsverdichtung und im schlimmsten Fall zu Ablehnung telemedizinischer Lösungen führt. Infolgedessen können SR ein Schlüsselfaktor bei der Überwindung einer schlechten Berichtsqualität aufgrund begrenzter Sprachkenntnisse sein, da hier standardisierte Simultanübersetzungen in mehrere Sprachen möglich ist [86, 91].

Neben der Befundqualität ist die Befunderstellungszeit im heutigen klinischen Alltag von zentraler Bedeutung. So wurde in diversen Publikationen nachgewiesen, dass die Befunderstellungszeit von strukturierten Befunden signifikant kürzer ist als die von Freitextbefunden [8, 9, 51, 74]. Dabei spielt jedoch die Komplexität des pathologischen Befundes eine große Rolle [42]. Während unauffällige Befunde überdurchschnittlich schnell mittels SR beschrieben werden können, scheinen komplexe pathologische Veränderungen tendenziell mehr Zeit in Anspruch zu nehmen. Ein möglicher Erklärungsansatz ist der hier notwendige Einsatz aufwendiger Freitextelemente [36, 83, 95]. Jedoch muss ein zeitlicher Mehraufwand hier immer vor dem Hintergrund der deutlich gesteigerten Befundqualität bewertet werden.

Von großer Bedeutung beim klinischen Einsatz wird die Integration von SR-Masken in die vorhandenen klinischen Informationssysteme sein, wie es bereits bei der standardisierten Befundung von pathologischen Veränderungen der Prostata und Mamma geschehen ist [67, 92].

### Kopf-Hals-Sonographie

Die Kopf-Hals-Sonographie stellt den klinischen Goldstandard in der bildgebenden Abklärung vieler Krankheitsbilder in der HNO dar [4, 6, 18, 33, 56, 71]. Dabei hat der Ultraschall nachgewiesenermaßen einen zentralen Stellenwert in der Primärdiagnostik, Verlaufskontrolle und Operationsplanung in der Kopf-Hals-Onkologie [1, 6, 56, 71]. Neben einer gründlichen Durchführung der Ultraschalluntersuchung selbst spielt die Befunderstellung eine zentrale Rolle und sorgt für einen hohen diagnostischen Qualitätsstandard, der die Basis für eine angemessene Therapieplanung und -durchführung darstellt. Während die konventionelle FTR mit einer niedrigen Intra- und Interrater-Reliabilität hinsichtlich Befundqualität und v. a. Vergleichbarkeit assoziiert ist, stellt die SR diesbezüglich einen vielversprechenden Ansatz dar [30, 76].

#### Auswirkungen auf die Aus- und Weiterbildung sowie die Qualitätssicherung

Während die praktische Durchführung von Ultraschalluntersuchungen einen gewissen Stellenwert in der Lehre und den Curricula der meisten medizinischen Hochschulen hat, werden Medizinstudierende mit der entsprechenden Befunddokumentation in aller Regel erst im Praktischen Jahr oder auch erst nach Approbation mit Beginn der Weiterbildung konfrontiert [114]. Der damit einhergehende Mangel an Ausbildung in der Befunderstellung resultiert vielfach in einem geringen Verständnis für die Befundstruktur, deren Inhalt, die korrekte Terminologie und in einer mangelnden Kenntnis der zu befundenden Strukturen. Dies steht in einem deutlichen Widerspruch zur Bedeutung des Befundes einer jeden diagnostischen Untersuchung und dessen Implikationen für medizinische Entscheidungsfindung und Therapieplanung. Der Befund stellt dabei die Essenz einer jeden Untersuchung dar, da er deren Inhalt, Beurteilung und Ableitungen daraus vermittelt. Zusätzlich ist er u. U. die Ausgangsbasis für Kontrolluntersuchungen, wie sie beispielsweise in der Kopf-Hals-Onkologie im Rahmen der Nachsorge nach festem Schema erfolgen [56, 57, 100].

Eine genaue Befundung ist dabei äußerst anspruchsvoll, da die Kopf-Hals-Region eine Vielzahl wichtiger Strukturen auf vergleichsweise geringem Raum aufweist. Auch die Interpretation der dreidimensionalen Topographie kann dabei herausfordernd sein [7]. Diese Schwierigkeiten werden durch einen Mangel an Standardisierung der Terminologie und Relevanz der anatomischen Strukturen verstärkt, die unerfahrenen Studierenden und Weiterbildungsassistenten möglicherweise unverständlich erscheinen. Darüber hinaus stellen Unklarheiten bei der Struktur eines Befundes, dessen Inhalt und Aufbau typische Hindernisse bei der Befunderhebung dar.

Daher kann SR als ein zentraler Baustein in der Verbesserung der Lehre im Studium und der ärztlichen Weiterbildung angesehen werden, da sie den Lernprozess begleitet und unterstützt, indem insbesondere noch unerfahrene Untersucher durch die Untersuchung und Befundung geführt werden, auf den relevanten Inhalt hingewiesen werden und ihnen die korrekte Terminologie an die Hand gegeben wird [53]. Dies wird durch diverse Studienergebnisse untermauert, die zeigen, dass die Anwendung einer SR zu weniger übersehenen pathologischen Veränderungen führt und die SR mit einer höheren diagnostischen Genauigkeit assoziiert ist [51, 63, 110].

#### Vergleichbarkeit

Trotz der vielen evidenten Vorteile der Sonographie bleibt die Interrater-Reliabilität ein zentrales Manko des Diagnostikums. Diese schnitt in Studien traditionell für die CT- und MRT-Diagnostik überlegen ab [40]. Jedoch existieren insgesamt nur wenig Daten zur Interrater-Reliabilität in der Kopf-Hals-Region und variieren in anderen Körperregionen stark nach Region und pathologischer Veränderung [108]. Dies kann zu großen Problemen bei der Abklärung von pathologischen Veränderungen im Weichgewebe der Kopf-Hals-Region und in der Folge zur Fehldiagnosen und intraoperativen Komplikationen führen [17]. Durch den Einsatz von SR wurde in der Folge im Rahmen von Studien eine hohe reproduzierbare Interrater-Reliabilität für die Kopf-Hals-Sonographie nachgewiesen, die sich mit bekannten Daten zu CT und MRT messen kann [24]. Dies ermöglicht, insbesondere unter Berücksichtigung der positiven Effekte auf Befundqualität und zeitliche Effizienz, eine genauere und insbesondere reproduzierbarere Ultraschalldiagnostik.

Neben all diesen Punkten existieren große nationale Unterschiede, welche Fachdisziplin den Kopf-Hals-Ultraschall durchführt. Während in vielen Ländern die Radiologie hier die dominierende Fachdisziplin ist, liegt dies im deutschsprachigen und skandinavischen Raum regelhaft in der Hand der HNO-Ärzte [109]. Dabei stellt insbesondere der sog. „physician-performed focused ultrasound“ einen zentralen Pfeiler bei der Operationsplanung minimal-invasiver Zugänge sowie auch für die etwaige intraoperative Sonographie dar [62]. Ohne eine entsprechende Expertise durch tägliche Routine ist in diesem Kontext keine sichere Anwendung möglich [85].

Es existieren multiple Publikationen zum Einfluss der SR auf die Befundqualität in verschiedenen Ausbildungsständen [25, 26, 28, 29]. Demnach verbessert die Anwendung von SR sowohl bei Medizinstudierenden, jungen und fortgeschrittenen Ärzten in Facharztweiterbildung sowie bei Fachärzten nachhaltig die Befundvollständigkeit, die verwendete Terminologie und auch die Befundung der pathologischen Veränderung. Zudem waren die Befunde signifikant besser lesbar als FTR. Die Befundqualität wurde von den Autoren als Summe aus Befundvollständigkeit, Terminologie, Lokalisation, Ausmaß und Form des pathologischen Befundes und Lesbarkeit definiert. Daraus ergab sich in allen Ausbildungsständen eine signifikant überlegene Befundqualität für die SR (*n* = 125; 93,1 vs. 45,6 %; *p* < 0,001) [29]. Im Rahmen weiterführender Analysen konnte für die wirtschaftlich zentrale Frage der für die Befunderstellung aufgewendeten Arbeitszeit gezeigt werden, dass die Zeitersparnis für SR im Vergleich zu FTR umso größer war, je früher SR in die Weiterbildung integriert wurde (R = 0,67; R^2^ = 0,449; *p* < 0,001) [29]. Die Zeitersparnis pro Befund lag dabei in frühen Phasen der Ausbildung bei bis zu 2,1 min pro Befund, was bei hoher Untersuchungsfrequenz zu einer relevanten Zeitersparnis pro Jahr führt. Diese Ergebnisse werden durch weitere Publikationen gestützt, die im longitudinalen Verlauf für die SR eine gleichbleibend sehr hohe Befundqualität zeigten, während die Befundqualität mittels FTR sich tendenziell verschlechterte. Zudem nahm die zeitliche Effizienz nur mittels SR im longitudinalen Verlauf stetig zu [28].

Darüber hinaus wurde gezeigt, dass die Anwenderzufriedenheit durch die Anwendung von SR signifikant steigt (visuelle Analogskale, VAS: 8,6 vs. 3,9; *p* < 0,001) [29]. Mögliche Gründe für die signifikant überlegene Benutzerzufriedenheit könnte der standardisierte SR-Algorithmus unter Einschluss einer vorgegebenen Terminologie sein, der die gängigen klinischen Standards und Leitlinien abbildet [79]. Dabei fördert die Redundanz der SR den Lernprozess, indem die Unterpunkte der Untersuchung in vorgegebener Reihenfolge abgefragt werden. Folglich zeigt sich, dass die Implementierung einer SR die Adhärenz in der Anwendung von Leitlinien und somit evidenzbasierter Medizin fördert [27]. Diese Ergebnisse befinden sich dabei im Einklang mit vorherigen Publikationen anderer Arbeitsgruppen, die eine Korrelation von SR mit qualitativ hochwertigen Befunden für verschiedene bildgebende Modalitäten zeigten [25, 26, 37, 48, 76, 94–98, 107, 117], ergänzen diese jedoch auch signifikant im Kontext der HNO sowie um Fragestellungen zur medizinischen Ausbildung.

#### Integration diagnostischer Parameter bei steigender klinischer Komplexität

In den letzten Jahren hat die Komplexität v. a. innerhalb der Kopf-Hals-Onkologie aufgrund der steigenden Anzahl von diagnostischen Parametern und Staging-Klassifikationen – wie der zuletzt eingeführten Differenzierung zwischen HPV-positiven und -negativen Oropharynxkarzinom in der 8. Auflage der TNM-Klassifikation [12] – deutlich zugenommen [50, 78, 84, 104]. Bei dieser spielt die genaue Anzahl von pathologischen Lymphknoten eine noch größere Rolle als bisher. Zudem besteht ein großes Interesse an bildgebenden Parametern zur Beurteilung des Ansprechens auf Radiochemotherapien [66, 90]. Um dieser steigenden Komplexität gerecht zu werden und um einen höchstmöglichen therapeutischen Standard zu gewährleisten, sind möglichst genaue, detaillierte und vergleichbare Befunde bei der Kopf-Hals-Sonographie von immenser Bedeutung. Während konventionelle Freitextbefunde, u. a. durch eine mangelnde Standardisierung der Terminologie und Befundstruktur, eine tendenziell niedrige Intra- und Interrater-Reliabilität in Bezug auf Befundqualität, Detailliertheit und Vergleichbarkeit aufweisen, stellen strukturierte Befunde einen vielversprechenden neuen Ansatz in der Befunderstellung dar [30, 76].

### Funktionelle endoskopische Nasennebenhöhlenchirurgie

Neben der Anwendung als Dokumentationswerkzeug in der Kopf-Hals-Sonographie existieren für die SR in der HNO publizierte Daten zur operativen Therapieplanung in der funktionellen endoskopischen Nasennebenhöhlenchirurgie („functional endoscopic sinus surgery“, FESS). Die FESS stellt den Goldstandard in der chirurgischen Therapie der chronischen Rhinosinusitis (CRS) dar [32, 46]. Die Evolution der FESS seit ihrer klinischen Etablierung in den 1980er-Jahren ist in großen Teilen dem technischen Fortschritt im zur Verfügung stehenden Equipment zu verdanken [19, 35, 54, 105]. Für die Planung von sicheren und erfolgreichen Operationen und insbesondere zur Vermeidung von potenziell katastrophalen Komplikationen wie Verletzungen der Orbita einschließlich des N. opticus, der vorderen Schädelbasis oder der A. carotis interna, ist eine hochauflösende, triplanare CT der NNH vor jedem Eingriff zwingend notwendig [26, 27]. Ferner dient die CT der NNH zur Festlegung der chirurgischen Resektionsplanung und zur Identifizierung des Zustands nach Voroperationen bzw. etwaiger anatomischer Normvarianten, die mit einem erhöhten intraoperativen Risiko einhergehen [41, 61, 64, 82, 118].

Das dreidimensionale Verständnis des Operationssitus stellt hierbei die Grundlage für das erfolgreiche Erlernen der Operationstechnik dar. Insbesondere seit Einführung der „powered instruments“, wie z. B. endonasale Shaver- oder Bohrersysteme, muss einer exakten Kenntnis der Anatomie Rechnung getragen werden, da diese „powered instruments“ bei Nichtbeachtung leicht zu schwerwiegenden Komplikationen, wie Verletzungen des Auges oder der vorderen Schädelbasis, führen können [15]. Darüber hinaus ist eine genaue Kenntnis des HNO-Arztes über die CT-morphologische Anatomie die Grundlage für die Anwendung von Navigationssystemen und für die interdisziplinäre erweiterte vordere Schädelbasischirurgie und könnte folglich durch vollständigere Resektionen zur Vermeidung von Revisionsoperationen beitragen [2, 106].

#### Auswirkungen auf die interdisziplinäre präoperative Diagnostik

Ein zentrales Problem in der präoperativen CT-basierten Planung vor FESS stellt die unterschiedliche Perspektive von HNO-Ärzten und Radiologen auf die zugrunde liegenden CT-Bilder dar [68, 89]. Während Radiologen sich i. Allg. mehr auf das Ausmaß und die sekundären Auswirkungen einer pathologischen Veränderung fokussieren, interessieren sich HNO-Ärzte insbesondere für anatomische Normvarianten und die knöchernen Begrenzungen, die als Landmarken für den chirurgischen Zugangsweg von Bedeutung sind [21]. Zudem fällt es sowohl radiologischen als auch HNO-ärztlichen Weiterbildungsassistenten häufig schwer zu entscheiden, welche anatomischen Strukturen einer dezidierten Beachtung bedürfen und wie diese adäquat zu beschreiben bzw. zu befunden sind [11, 112]. In der Folge existieren in vielen Ausbildungskliniken Checklisten in Papierform, anhand derer wichtige anatomische Strukturen der Reihenfolge nach identifiziert und befundet werden können. Da solche Checklisten meist nur in analoger Form existieren und folglich dem Weiterbildungsassistenten kein Feedback liefern können, werden sie häufig nach Implementierung nur unregelmäßig verwendet, sodass keine Integration in den klinischen Arbeitsablauf stattfindet [47]. Jedoch existieren wenige Erkenntnisse zum Einfluss einer strukturierten Operationsplanung in der präoperativen Phase. Dies steht im Gegensatz zur zentralen Bedeutung einer präzisen Befundung der präoperativen Bildgebung sowie einer subtilen Planung des operative Zugangs und Ausmaßes [77]. Zudem kann eine detaillierte präoperative Planung das Selbstvertrauen und die Lernkurve von Weiterbildungsassistenten positiv beeinflussen und somit zu einer reduzierten Inzidenz von Komplikationen und einer gesteigerten Wirtschaftlichkeit beitragen [45, 69].

Zur Anwendung von SR im Rahmen der radiologischen CT-Diagnostik und der FESS-Planung existieren im Wesentlichen 2 Studien. In diesen wurde die Anwendung im klinischen Alltag sowie im Rahmen eines FESS-Operationskurses untersucht [5, 27].

Im Rahmen der klinischen Routine wurden bei 30 konsekutiven Patienten, die zur FESS anstanden, die vorliegenden CT-Untersuchungen erneut mittels einer dezidierten SR-Maske zur CT der NNH erneut befundet und mit den Befunden aus der klinischen Routine bezüglich chirurgisch und radiologisch relevanter Parameter verglichen [27]. Ferner erfolgte die Erstellung einer schriftlichen Operationsplanung durch den eingeteilten Weiterbildungsassistenten, sowohl konventionell mittels FTR als auch mittels SR. Es zeigte sich, dass die CT-Befunde mittels SR deutlich vollständiger mit Hinblick auf alle evaluierten Parameter waren (84,4 vs. 22,0 %; *p* < 0,001).

#### Auswirkungen auf die Operationsplanung

Zudem konnten die Weiterbildungsassistenten mittels SR signifikant häufiger relevante pathologische Veränderungen als auch anatomische Besonderheiten identifizieren (97,0 % vs. 39,4 %; *p* < 0,001). Insbesondere führt die Implementierung einer strukturierten Operationsplanung zu einer signifikanten Steigerung der Berücksichtigung von potenziell gefährdeten anatomischen Strukturen, wie der Lamina papyracea, der vorderen Schädelbasis und deren Asymmetrien entsprechend der Keros-Klassifikation. Zudem waren der Zeitbedarf zur Erstellung der entsprechenden Operationsplanungen sowie die Benutzerzufriedenheit mittels SR deutlich besser.

In einer weiteren Studie wurden 15 Teilnehmer eines FESS-Kurses um Erstellung einer CT-basierten FESS-Operationsplanung sowohl mittels FTR als auch SR gebeten. Bei der Auswertung zeigte sich bei deutlich höherem Zeitbedarf ebenfalls eine sicherere Identifikation relevanter pathologischer Veränderungen und anatomischer Varianten sowie eine höhere Benutzerzufriedenheit für die strukturierte Herangehensweise.

In der Folge reduziert eine verbesserte Dokumentation bzw. Operationsplanung am Beispiel der SR, gerade in Abteilungen mit hohen Fallzahlen und bei Weiterbildungsassistenten im Lernprozess, das Risiko, dass atypische kritische anatomische Strukturen nicht identifiziert werden und sich somit die Gefahr für intraoperative Komplikationen erhöht. Ferner erhöht eine konsequente und ausführliche präoperative Dokumentation die medikolegale Sicherheit des behandelnden Arztes. Zudem könnte eine gründliche Operationsplanung dazu beitragen, Revisionseingriffe zu vermeiden.

### Neurootologie

Ein weiterer evaluierter Arbeitsbereich der SR ist die interdisziplinäre Abklärung von Schwindelbeschwerden [60]. Solcherlei Beschwerden stellen mit bis zu 4 % aller Patienten in Notaufnahmen einen häufigen Konsultationsgrund dar [38, 43, 73, 80]. Von zentraler Bedeutung in der Abklärung ist die Differenzierung zwischen den größtenteils harmlosen, jedoch in 5–12 % der Fälle lebensbedrohlichen Ursachen [20, 44, 93]. Letztere gilt es ohne Verzögerung und somit Kompromittierung des diagnostischen Zeitfensters zu selektionieren und sie der korrekten Diagnostik und Therapie zuzuführen.

Im Gegensatz zu den gut definierten neurootologischen Krankheitsbildern stehen Schwindelbeschwerden mit komplexer, multifaktorieller Genese, die große Ansprüche an die interdisziplinäre Versorgung stellen. Dabei stellt die strukturierte und standardisierte Anamnese die Basis für eine problemorientierte und kosteneffiziente Diagnostik dar. Diagnostisch spielt in der HNO-ärztlichen Abklärung die neurootologische Funktionsdiagnostik eine tragende Rolle, deren Sensitivität der radiologischen Schnittbildgebung häufig überlegen ist [99]. Aufgrund der regelhaft interdisziplinären Abklärung ist eine adäquate intersektorale sowie interdisziplinäre Kommunikation unabdingbar. Grundlage hierfür ist insbesondere eine hohe Befundqualität. Diese reduziert nachweislich potenzielle Informationsverluste, aber auch wiederholte Untersuchungen und Therapieverzögerungen [72]. Ferner erhöht die problemlose und ausführliche Kommunikation nachhaltig die Zuweiserzufriedenheit und verbessert die intersektorale und auch die fachübergreifende Zusammenarbeit [5, 25, 27, 49, 116].

Die Anwendung von SR als Dokumentations- und Kommunikationsmedium in der interdisziplinären Schwindelabklärung wurde durch Lasrich et al. untersucht [60]. Dabei wurden bei 100 konsekutiven Patienten anhand der vorliegenden Anamnese sowie der Rohdaten der neurootologischen Funktionsdiagnostik SR erstellt und mit den Arztberichten aus der klinischen Routine verglichen sowie die Zuweiserzufriedenheit erhoben. Dabei zeigte sich eine signifikant erhöhte Vollständigkeit hinsichtlich Anamnese, neurootologischer und audiologischer Funktionsdiagnostik. Zudem erhöhte sich mittels SR nachhaltig die Zufriedenheit der zuweisenden Ärzte.

Diese gesteigerte Qualität der Dokumentation und Kommunikation kann dabei zur Ökonomisierung der diagnostischen Ressourcen beitragen. Diese sog. Testökonomie durch an die Beschwerden exakt angepasste Diagnostik unterstützt nachweislich die Diagnosefindung, reduziert Enttäuschung sowohl aufseiten der Patienten als auch der behandelnden Ärzte und verbessert die Compliance [22]. Die könnte insbesondere bei Weiterbildungsassistenten die Begeisterung für das komplexe Gebiet der Neurootologie steigern und der zuletzt sinkenden Anzahl von Spezialisierungen in diesem Teilgebiet entgegenwirken [39].

## Ausblick

Als Ausblick für die SR werden derzeit Lösungen für alle Teilaspekte der HNO sowie der Kopf- und Halschirurgie entwickelt. Von zentraler Bedeutung sind dabei insbesondere Anamnese, Diagnosen, Therapien und die Erfassung von Outcomes als zentrales Element der Qualitätssicherung. Konkrete Projekte umfassen zum einen um Dokumentationen von allgemeinen HNO-ärztlichen Untersuchungen einschließlich der Möglichkeit zur digitalen Befunddokumentation anhand von Skizzen. Ferner soll die Anwendung im Rahmen von strukturierten Operationsberichten überprüft werden. Ein weiteres Anwendungsgebiet stellt die Befundberichterstellung von Schlaflabordiagnostiken nach den Vorgaben der Deutschen Gesellschaft für Schlafforschung und Schlafmedizin e. V. dar, die im Rahmen von klinischen Studien evaluiert wird [3]. Darüber hinaus existieren drittmittelgeförderte Projekte zur integrierten, fachübergreifenden Versorgung von Patienten mit Polyposis nasi, anhand derer viele Erkenntnisse zur interdisziplinären Anwendung sowie wissenschaftlichen Auswertbarkeit zu erwarten sind.

Schlussendlich wären multizentrische Projekte zur Evaluation der Kombination aus KI und SR sowie zur wissenschaftlichen Auswertbarkeit von strukturierten Befunden von großem Interesse.

## Fazit für die Praxis

Die Anwendung strukturierter Befunderhebung (SR) hat in den Teilgebieten der HNO

- Kopf-Hals-Sonographie,
- interdisziplinäre Planung von Nasennebenhöhlenoperationen,
- interdisziplinäre neurootologische Abklärung von Schwindelbeschwerden

einen positiven Einfluss auf

- die Befundqualität und -vollständigkeit,
- den longitudinalen Lerneffekt,
- die zeitliche Effizienz,
- die Interrater-Reliabilität,
- die Benutzer- und Zuweiserzufriedenheit sowie die interdisziplinäre Kommunikation.
